# A novel cancer preventative botanical mixture, TriCurin, inhibits viral transcripts and the growth of W12 cervical cells harbouring extrachromosomal or integrated HPV16 DNA

**DOI:** 10.1038/s41416-020-01170-3

**Published:** 2020-12-01

**Authors:** Linda Saxe Einbond, Jing Zhou, Hsan-au Wu, Emeka Mbazor, Guiyun Song, Michael Balick, James A. DeVoti, Stephen Redenti, Mario R. Castellanos

**Affiliations:** 1 The New York Botanical Garden, Bronx, NY 10458 USA; 2 Lehman College and the Graduate Center, City University of New York, New York, NY 10468 USA; 3 Columbia University College of Physicians and Surgeons, New York, NY 10032 USA; 4 R&D Department, Professional Compounding Centers of America, Houston, TX 77099 USA; 5 The Feinstein Institutes for Medical Research, Northwell Health, Manhasset, NY 11030 USA; 6 Innovene Therapeutics, Brooklyn, NY 11230 USA; 7 Staten Island University Hospital, Northwell Health, Staten Island, NY 10305 USA

**Keywords:** Gynaecological cancer, Cancer, Drug development, Drug development, Microarrays

## Abstract

**Background:**

The phytochemical mixture TriCurin (curcumin, epigallocatechin gallate (EGCG) and resveratrol) eliminates human papillomavirus (HPV) (+) cancer cells in vitro and in vivo. In this study, we further evaluate TriCurin.

**Methods:**

The activity of TriCurin and its individual compounds was assayed on W12 cells, derived from a cervical precancer containing episomal and integrated HPV16 DNA, using MTT (3-(4,5-dimethylthiazol-2-yl)-2,5-diphenyltetrazolium bromide) assays, microscopy and reverse transcription-polymerase chain reaction (RT-PCR), and on HeLa cells by gene expression analysis. The stability and toxicity of TriCurin microemulsion were tested in an organotypic cervical tissue model.

**Results:**

TriCurin and its individual compounds inhibit the growth of W12 cells, episomal, type 1 and 2 integrants; the relative order of activity is TriCurin, EGCG, curcumin, or resveratrol. RT-PCR shows that TriCurin activates p53 and suppresses HPV16 mRNAs E1, E2, E4, E6 and E7 at 24 h in W12 cells. Gene expression analysis shows that TriCurin activates pro-apoptotic genes and represses anti-apoptotic genes in HeLa cells. TriCurin in a microemulsion is stable and non-toxic to cervical tissue. The combination of TriCurin and tanshinone IIA exhibits additional synergy against HeLa cells.

**Conclusions:**

TriCurin, and the combination of TriCurin with tanshinone IIA, are effective against HPV (+) cells. The phytochemical mixture, in the microemulsion-based cream, is a promising therapeutic for the prevention and treatment of cervical cancer.

## Background

Despite advances, human papillomavirus (HPV) continues to cause significant morbidity and mortality, as evidenced by 570,000 cases of cervical cancer and 311,000 deaths globally in 2018.^1^ Cervical cancer is the fourth leading cause of cancer death in women; the mortality rates are highest in low-income countries. Although the incidence has been considerably reduced in developed regions, such as the United Kingdom, millions of women are identified to harbour high-risk HPV infection. About 10% of HPV (+) women will progress to high-grade cervical dysplasia within 2 years.^2^ Based on the number of HPV-infected women worldwide, the global prevalence of pre-cancerous lesions of the cervix is estimated to be 30–40 million.^3^ Surgical and ablative interventions are the only available options to treat pre-cancerous lesions of the cervix once they develop. However, these modalities increase the potential for future adverse complications during pregnancy.

It is imperative to develop a therapeutic agent for high-risk HPV infections and the associated lesions. The HPV vaccines are effective at preventing disease, but access to these vaccines may be limited in developing countries and these vaccines do not benefit women who are already HPV-infected. Studies have shown that curcumin,^4^ tanshinone IIA,^5–7^ ellagic acid,^8^ epigallocatechin gallate (EGCG)^9^ and TriCurin^10^ inhibit the growth of cervical cancer cells. We further show that these plant-derived polyphenols, either alone or in combination, can inhibit HPV genes and eliminate HPV (+) cancer cells (L.S. Einbond et al., manuscript in preparation). These compounds target multiple cellular pathways, are available in developing countries, are inexpensive and thus provide an opportunity for drug development.

HPVs are small non-enveloped double-stranded DNA viruses that contain several early viral genes coding for proteins E1, 2, 4, 5, 6 and 7, and two late proteins L1 and L2. The viral proteins E1 and E2 control viral amplification. E6 represses p53 and activates telomerase, while E7 promotes degradation of the Rb protein family; both actions result in increase in cell growth and viral replication. E4 and E5 facilitate viral replication. L1 is the major capsid protein.^11^

A role for high-risk HPV’s in the development of cervical cancer has been shown by epidemiological and laboratory studies. Establishing and maintaining the malignant state requires the overexpression of high-risk HPV E6 and E7 proteins, which can occur if HPV DNA becomes integrated disrupting the regulatory effects of E2 on these two oncogenes.^12^

TriCurin is a combination of three purified plant-derived polyphenols mixed in specific ratios, curcumin, from the spice turmeric (*Curcuma longa* L., Zingiberaceae), resveratrol, from grapes (*Vitus vinifera* L., Vitaceae) and EGCG, from green tea (*Camellia sinensis* (L.) Kuntze, Theaceae) (structures: Supplementary Fig. S1A–C). The optimised molar ratio of curcumin to EGCG to resveratrol is 32:8:100, which produces a potent synergistic mixture. Previous studies indicate that developing mixtures with antioxidant activity can stabilise plant-derived polyphenols, such as curcumin, that rapidly degrade in an aqueous environment.^10^ TriCurin was designed to have maximised anti-cancer activity, improved stability and increased cellular uptake. Previous studies indicated that TriCurin repressed the expression of E6 and E7 and activated the expression of p53 in HeLa cells.^10^ In an HPV (+) chimeric mouse model of cervical cancer, both TriCurin and curcumin suppressed E6 and induced p53, acetyl-p53 and caspase-3. The response to TriCurin was several folds higher than the response to curcumin, while having better stability in an aqueous environment.^10^ In addition, TriCurin inhibited growth, clonogenic survival and tumoursphere formation in head and neck squamous cell carcinoma (HNSCC), and inhibited tumour growth in a preclinical model of HPV (+) HNSCC.^13^

To further evaluate the chemo-preventative potential of TriCurin, we explored the growth inhibitory effects on a unique set of cell lines derived from a cervical pre-cancerous lesion (CIN grade 1).^14^ The subclone 20850 contains ~5–100 extrachromosomal copies of the HPV16 genome. After culture in vitro, the W12 cells generated the clones 20822 and 210402 containing 3 and 5 HPV16 genomes, respectively, that are recombined with the cellular DNA (type 1 integrated clones). In addition, the W12 cells generated clones 20861 and 20862, which contain 30 or 60 recombined viral genomes (viral genomes plus junction fragments), respectively, most of which are repeated in tandem or as concatemeres (type 2 integrated clones).^14^ The W12 type 1 and 2 clones resemble the HPV DNA integration state of cancer-derived cells lines, SiHa (1–2 HPV16 copies) and CaSki (500 HPV16 copies), respectively.^14^

To advance towards the clinical setting, in this study we optimise the TriCurin mixture and develop a microemulsion-based cream appropriate for use in a future clinical trial. Since combination of phytochemicals, in previous studies, produced the most potent and stable mixtures, we tested the growth inhibitory effects of various TriCurin formulations combined with active phytochemicals, including turmeric (with 90% or 95% curcuminoids) and tanshinone IIA. Tanshinone IIA is the most abundant diterpene quinone present in the root of red sage (*Salvia miltiorrhiza* Bunge, Lamiaceae).

We validated the clinical potential of these phytochemical mixtures using W12 cells in vitro and examined the effects of TriCurin on the early stages of HPV replication, the expression of p53 and HPV16 E1, E2, E4, E6 and E7 viral transcripts. Our findings indicate that TriCurin, containing turmeric (95% curcuminoids), strongly inhibits the growth of W12 cells containing either episomal or integrated HPV16 DNA. Genome-wide analysis indicates that TriCurin reverses HPV-induced gene alterations. The phytochemical mixture in the microemulsion-based cream is a promising therapeutic candidate to prevent and treat cervical cancer.

## Methods

### Materials

All solvents and reagents were reagent grade; H_2_O was distilled and deionised. Tanshinone IIA was purchased from Sigma (St. Louis, MO) and was dissolved in ethanol (100%). The two turmeric preparations contained the curcuminoids curcumin, desmethoxycurcumin, and bis-demethoxycurcumin as follows: (1) *Naturex #140500* (Avignon, France); 95% curcuminoids (high-performance liquid chromatography, Supplementary Fig. S1D), (2) *Curcumin powder 95%*, 89.88% curcuminoids and volatile oils of turmeric rhizome (Dolcas Biotech LLC; PCCA, Houston, TX). Both were dissolved in dimethylsulfoxide (DMSO) (Sigma; St. Louis, MO), prior to addition to cell cultures. Resveratrol (99.0%; Jiaherb) and EGCG (95.3%; Taiyo Green Power Co., Ltd) were obtained from PCCA (Houston, TX), and were dissolved in ethanol. All solutions were sonicated for 10 min. The source of the compounds for the gene expression studies was as follows: curcumin (≥98% curcuminoid content) (CAS number 458-37-7) (Thermo Fisher Scientific, NJ, USA; prepared by Acros Organics for Thermo Fisher; stored at room temperature under nitrogen), (−)-epicatechin gallate, and resveratrol (Thermo Fisher).

### Preparation of TriCurin

To prepare TriCurin, the following volumes of stock solutions were added to 5 mL Dulbecco’s modified Eagle’s medium (DMEM): resveratrol, 2.5 µL of 200 mM stock; EGCG, 1.6 µL of 25 mM stock; curcumin (turmeric (90% curcuminoids), except where noted), 4 µL of 40 mM stock; mixed briefly after each addition, and sonicated for 10 min. The concentration of DMSO is 1/1250 in TriCurin and this is further diluted when the cells are treated, DMSO < 0.08%.

### Cell cultures and tissues

HeLa human cervical cancer cells were a kind gift of Dr. Bettie Steinberg (Northwell Health, NY; October 11, 2016) and were obtained from ATCC (Manassas, VA) (combination assays). MDA-MB-453 (ER‐negative, Her2-overexpressing) cells were obtained from ATCC (Manassas, VA). HeLa and MDA-MB-453 cells were grown in DMEM (Gibco-BRL Life Technologies, Inc., Rockville, MD) containing 10% (v/v) foetal bovine serum (FBS) (Gibco-BRL), plus Pen-Strep (Gibco). MCF7 and MCF7/Her2–18 (MCF7 cells transfected with a full-length Her2 cDNA coding region, 45-fold increase Her2)^15^ were kindly sent to us by Dr. Dennis Slamon (Los Angeles, CA). These cells were cultivated in RPMI supplemented with 10% FBS plus glutamine (1%) and PSF (penicillin G-streptomycin-fungizone solution) (1%) (Gibco). Cells were maintained at 37 °C, 5% CO_2_ and used at passages 1–4.

The online ATCC brochure Maintaining High Standards in Cell Culture describes the accessioning process, during which all ATCC cell lines undergo authentication tests.^16^

Human EpiVaginal tissues were purchased from MatTek (Ashland, MA, USA).

### Cell culture: W12 cells

W12 subclones (20850W12E clone; 20822 and 201402 W12I clones with type 1 integration pattern; 20861 and 20862 referred to as type 2 integrants) and J2 3T3 mouse embryo fibroblasts were a kind gift of Dr. Paul Lambert (University of Wisconsin, Madison, WI; November 28, 2016). W12 clones were cultured, as previously described.^14^

### Proliferation assay

Cells were seeded in 96-well plates at 1000 cells per well. Twenty-four hours after plating, phytochemicals were added and cells were incubated for 72, 96 or 120 h. The sensitivity of the various cell lines to herbal agents was assayed using the MTT (3-(4,5-dimethylthiazol-2-yl)-2,5-diphenyltetrazolium bromide) assay, as previously described,^17^ or the EZQUANT assay. For the EZQUANT assay, absorbance was read at 420 nM and corrected for the background value.

### Calculating the combination index

To assess possible synergistic effects, cells were treated with all combinations of four concentrations of each of the agents tested and a solvent control.^18^ The median effect principle was used to analyse the combination assays.^18^ We used variable ratios of drugs and assumed mutually exclusive equations to determine the combination index (CI).^18^ Half-maximal inhibitory concentration (IC_50_) values determined from the combination graphs were used to calculate CI values; for agents 1 and 2: CI = [IC_50_ (agent 1 + agent 2)/(IC_50_ (agent 1 alone)] + [IC_50_ (agent 2 + agent 1)/IC_50_ (agent 2 alone)]. CI and its corresponding effect are as follows: >1.3, antagonism; 1.1–1.3, moderate antagonism; 0.9–1.1, additive effect; 0.8–0.9, slight synergism; 0.6–0.8, moderate synergism; <0.6, synergism.

### RNA extraction and quantitative real-time PCR

Cells were seeded and cultured on 12-well plates at 2 × 10^5^ cells per well. Each drug concentration was performed with triplicates. At 0, 6 and 24 h, cells were collected, and total RNA extraction was performed using Trizol reagent (Thermo Fisher Scientific, USA). RNA concentration was measured using NanoDrop 2000. cDNA was synthesised using ProtoScript® First Strand cDNA Synthesis Kit (NEB Biolabs). GAPDH (glyceraldehyde 3-phosphate dehydrogenase) was used as a housekeeping gene control for mRNA analysis. Quantitative real-time PCR (qPCR) was performed using SYBR GreenER™ qPCR SuperMix (Life Technologies) on a Bio-Rad system. Fold-change 2−∆∆C method was used to calculate mRNA abundance. All primers are listed in Supplementary Table S1. Student’s *t* test was run to calculate significance of triplicates.

### Gene expression analysis

Gene expression analysis was performed, as previously described.^19^ HeLa cells were treated with TriCurin (32 µM) or its individual phytochemicals, curcumin (32 µM), EGCG (8 µM), resveratrol (100 µM) or control (DMSO) for 8 h. DMSO in all the results refers to the control condition, which has DMEM culture medium containing 0.28% of DMSO. The cells (1.5 × 10^5^ per well, in 24-well plates) were treated in triplicate in serum-free DMEM containing insulin-transferrin-sodium selenite (ITS). At the end of treatment, 200 µL of RLT buffer (Qiagen RNA isolation kit) was added to each well and samples were frozen at −80 °C. The *p* values are calculated using ANOVA (analysis of variance) KaleidaGraph with post hoc tests performed using Tukey. Differentially expressed genes were selected from the KEGG pathway database (https://www.genome.jp/kegg/). Data were analysed using the Genesifter software (VizX Labs, Seattle, WA, USA). Data were extracted to show genes significantly affected by TriCurin compared to individual compounds (C, E or R). All RefSeq and UniProt references were taken from genecards.org.

### Microscopy

The effect of phytochemicals on human cervical cells was examined by microscopy using the NIS Elements software paired with a Nikon Eclipse TI-S microscope. Images were captured using bright-field channel at magnifications of ×10, ×20 and ×40; EpiVaginal cervical tissues, ×100.

### Preparation of TriCurin-Enpen cream and stability testing by biological assay

Manufacture of TriCurin cream was done at PCCA (Houston, TX, USA). TriCurin components included curcumin (BCM-95, Dolcas Biotech, LLC), epigallocatechin gallate (Sunphenon EGCG, Taiyo Green Co., Ltd), resveratrol (ReserveNature, JIAHERB). A proprietary oil-in-water microemulsion, called Enpen (developed by PCCA and Innovene Therapeutics), was used to make 20% TriCurin-Enpen cream (w/w) (Supplementary Fig. S2). Compounds were mixed in Enpen to achieve curcumin 160 mM, EGCG 40 mM, and resveratrol 500 mM; this produces a formulation with the synergistic molar ratio of 4:1:12.5, respectively. The concentration of this mixture was designated 160 mM TriCurin-Enpen. The cream was stored and protected from light at 4 °C. To test the cream’s stability, a biologic assay was developed in which the cytotoxic effects of TriCurin in Enpen were evaluated over time against HeLa cells (times 0, 7 days and 3.5 months). On the day of testing, 160 mM TriCurin-Enpen was diluted with DMEM to obtain 320 µM (w/v), and Enpen cream alone was diluted with DMEM to obtain 320 µM (w/v). Serial dilutions were further made for a dose–response analysis. HeLa cells were cultured in DMEM medium containing 10% FBS, 100 U/mL penicillin and 100 µg/mL streptomycin in a 37 °C humidified incubator and an atmosphere of 5% CO_2_, in a T75 flask. Two thousand cells were seeded in 96-well plates and were cultured overnight. After 96 h, the effect of the drug at different concentrations on the plated cells was tested by MTT assay. The absorbance was recorded at 570 nm. Background correction was achieved using separate wells without cells, but containing all other reagents.

### Evaluation of the safety and toxicological profile of TriCurin-Enpen cream: TriCurin-Enpen on human EpiVaginal tissues

To evaluate the safety and toxicity of Enpen and TriCurin-Enpen, in vitro testing was done using organotypic human vaginal-ectocervical (VEC) tissue (EpiVaginal, MatTek, Ashland, MA).^20^ Endpoints of interest are tissue viability and histology. EpiVaginal tissues were pre-incubated with 5 mL medium at 37 °C, overnight. The next day, 100 µL of each test material was applied, in triplicate, on EpiVaginal tissue for 24 h. The receptor medium is 1000 µL in 6-well plates (Supplementary Fig. S3: illustration of tissue well; 3D cervical tissue model).

To evaluate if TriCurin-Enpen and Enpen creams are non-toxic on Human EpiVaginal tissues, EpiVaginal cervical tissues were treated with (1) Enpen cream or two positive control creams known to be toxic Gynol II (nonoxynol-9 (3%) and 1% Triton X-100) and (2) TriCurin-Enpen and Enpen creams compared to Vagisil (benzocaine 5%, resorcinol 2%) and Gynol II (nonoxynol-9 (3%)), for 24 h.

For histologic examination, EpiVaginal cervical tissues were treated with either Control (negative control), TriCurin-Enpen, Gynol II or Vagisil for 4 h. Entire EpiVaginal tissue implants were fixed in 10% formalin (overnight at room temperature), paraffin-embedded, sectioned (5–6 µm thick) using a microtome and stained with haematoxylin and eosin, according to standard procedures.

### Statistical analysis

We performed two-tailed Student’s *t* test assuming two samples with equal variance, and two-factor analysis of variance (ANOVA)-based *t* test for two different means; comparison standard error (SE) was estimated by ANOVA; theoretically, the ANOVA based *t* test is more accurate than the simple Student’s *t* test. Statistical significance was defined as *p* < 0.05 (Supplementary information).

## Results

### Growth inhibitory activity of TriCurin and its individual compounds on W12 cell lines

To gain insight to the action of TriCurin (Cur:EGCG:Res; 32:8:100) and its individual compounds, we assayed the growth inhibitory effect of TriCurin on W12 cervical cells, which contain copies of HPV16 in different states of integration (five W12 cell lines), as well as 3T3 feeder cells.

#### Effects of TriCurin and its individual compounds on 3T3 feeder cells

**Fig. 1:**
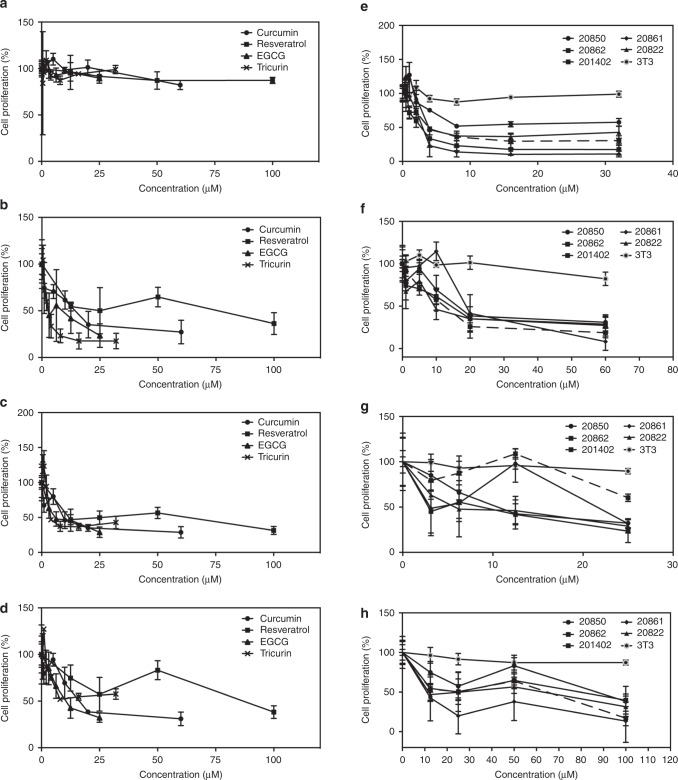
Growth inhibitory activity of TriCurin and its individual compounds on W12 cells derived from a cervical pre-cancerous lesion. **a**–**d** 3T3 and W12 cells: **a** 3T3; **b** 20862; **c** 20822; **d** 20850; **e**–**h** TriCurin and its individual compounds: **e** TriCurin; **f** EGCG; **g** curcumin; **h** resveratrol. Cells were exposed to increasing concentrations of agents for 120 h and the number of viable cells was determined by the MTT assay. The *p* value for TriCurin (2 μM) for 20850 vs. 20862 cells is 0.0350.

The W12 cultures contained irradiated 3T3 (mitomycin C-treated J2 3T3 feeder) cells that are metabolically active, but not replicating. The ratio of feeder to W12 was ~1:20 to 1:100 (for 20861, 20862, 20822 and 201402 cells); for the 20850 cells, the ratio was ~1:10 to 1:50. The feeder cells exhibited resistance to TriCurin and its individual compounds (Fig. 1a). Thus, the feeder cells have little effect on the assay since they represent a small percent of the cells and possess resistance to the phytochemicals.

#### Effects of TriCurin and its individual compounds on W12 cell lines

The growth inhibitory effects of TriCurin were compared on the W12 cell lines. The IC_50_ values on 20861 cells were as follows (Fig. 1b): TriCurin, 0.9 μM; EGCG, 3 μM; resveratrol, 10 μM; curcumin, 14 μM. On 20822 cells, the relative activity was as shown in Fig. 1c: TriCurin, 3.9 μM; EGCG, 5.8 μM; resveratrol, 11.8 μM; curcumin, 9.42 μM. On 20850 cells, the relative activity was as shown in Fig. 1d: TriCurin, 8.2 μM; EGCG, 10.5 μM; curcumin, 16.3 μM; resveratrol, 53.7 μM. On 20862 cells, the relative activity was: TriCurin, 2.7 μM; EGCG, 8.8 μM; curcumin, 14.4 μM; resveratrol, 25 μM (data not shown).

Thus, the order of activity of TriCurin and its individual compounds on W12 cells is: TriCurin, EGCG, resveratrol, or curcumin. EGCG and TriCurin are about equally active on 20850 cells, which contain episomal HPV16 genomes.

The lowest concentrations of TriCurin that induced significant growth inhibitory effects on W12 cells are as follows: 8 μM on 3T3 (*p* = 0.0456); 4 μM on 20850 (*p* = 0.0108); 0.5 μM (*p* = 0.0245) or 4 μM (*p* = 1.68E − 05) on 20822; 1 μM on 20862 (*p* = 0.0106). The lowest concentrations of TriCurin and its individual compounds that induced significant effects on 20850 cells are: TriCurin (4 μM), *p* = 0.0108; curcumin (20 μM), *p* = 0.00171; EGCG (12.5 μM), *p* = 0.0252; resveratrol (25 μM), *p* = 0.0173.

#### Comparison of the effects of TriCurin and its individual compounds on W12 cell lines

The growth inhibitory effects of TriCurin and its individual compounds were assayed on the W12 cell lines. For TriCurin, the IC_50_ values were as follows (Fig. 1e): 20861, 0.9 μM; 20862, 2.7 μM; 201402, 3.8 μM; 20822, 3.9 μM; 20850, 8.2 μM. Analysis by microscopy indicated treatment of 20850 cells with TriCurin inhibited proliferation and resulted in degeneration of the cells, characteristic of apoptosis (data not shown).

Regarding the individual phytochemicals, for EGCG, the IC_50_ values were as follows (Fig. 1f): 20861, 3 μM; 20822, 5.8 μM; 20862, 8.8 μM; 20850, 10.5 μM; 201402, 27 μM. For curcumin, the IC_50_ values were as follows (Fig. 1g): 20822, 9.42 μM; 201402, 12 μM; 20861, 14 μM; 20862, 14.4 μM; 20850, 16.3 μM. For resveratrol, the IC_50_ values were as follows (Fig. 1h): 20861, 10 μM; 20822, 11.8 μM; 201402, 25 μM; 20862, 25 μM; 20850, 53.7 (88) μM.

For TriCurin, in this experiment, the order of sensitivity of the three cell types is as follows: 20861, 20862, 201402, 20822, 20850 and 3T3. The lowest concentrations of TriCurin that induced significant effects on 20850 vs. the other cell lines are: TriCurin (4 μM) on 20850 vs. 3T3 (*p* = 0.00101); TriCurin (4 μM) on 20850 vs. 20822 (*p* = 2.06E − 06); TriCurin (2 μM) on 20850 vs. 20862 cells (*p* = 0.0350).

### Reverse transcription-polymerase chain reaction (RT-PCR) analysis of the relative level of HPV16 and p53 mRNAs in the W12 cells

To generate adequate cells for the PCR experiments, the 20850 cells were passaged two more times than the 20822 and 20862 cells. To lend insight into the state of the viral DNA, we assessed the relative level of the HPV16 genes in the W12 cell lines at 0 time. The primers used are listed in Supplementary Table S1. The results are displayed in Supplementary Table S2.

The relative level of expression of the genes in 20850 cells is set to one. The level of expression of the early gene *E1* is ten or more times higher in 20850 (1) vs. 20822 (0.07) and 20862 (0.19) cells. This is consistent with an episomal state for the viral genome in the 20850 cells. E2 mRNA is also reduced in 20822 and 20862 vs. 20850 cells, about 2-fold. The level of E4 and E7 is reduced (~2-fold) in 20822 cells and slightly reduced in 20862 cells. The level of E6 is reduced in 20822 cells and increased in 20862 cells. For the tumour suppressor gene p53, the level is many folds higher in 20850 vs. 20862 (0.16) or 20862 (0.38) cells. Thus, the 20850 cells contain the highest level of viral mRNAs, with the exception of E6 in 20862 cells. The differences between the relative levels of p53 and HPV16 mRNAs in the three cell types (20850 vs. 20822; 20850 vs. 20862; 20822 vs. 20862) at 0 time are all significant (*p* < 0.01).

### RT-PCR analysis of the effects of TriCurin on the expression of p53 and HPV16 mRNAs in W12 cells

**Fig. 2:**
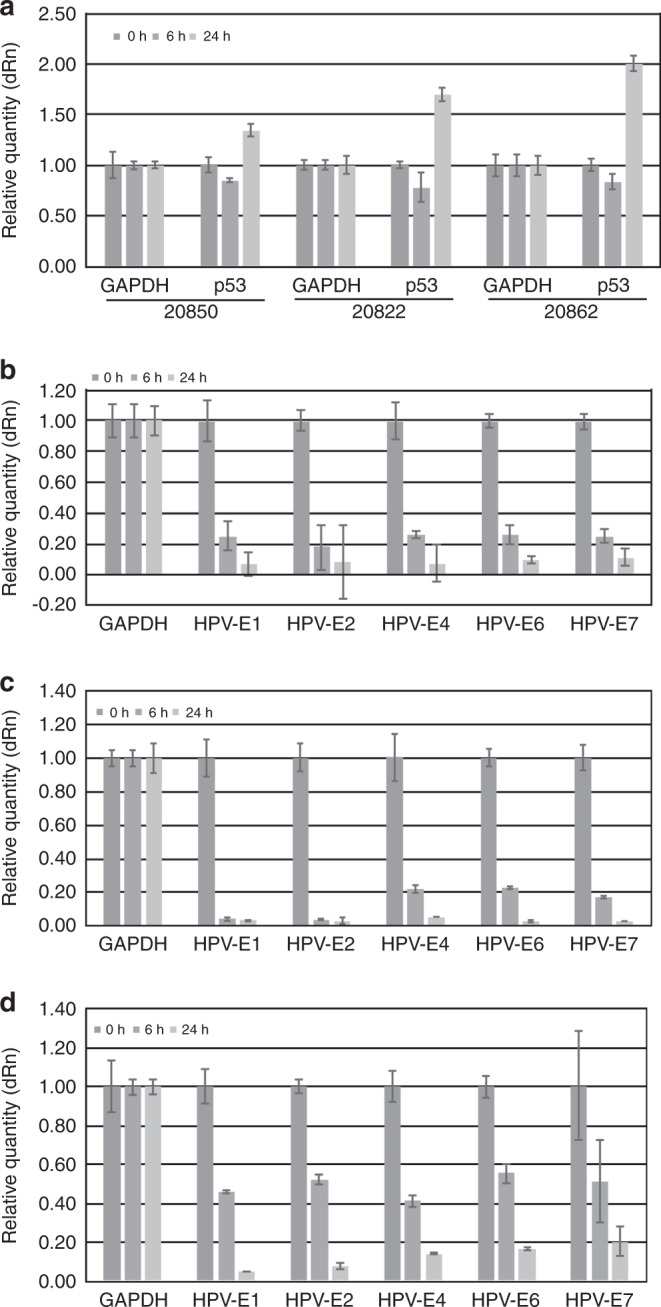
RT-PCR analysis of RNA obtained after treating W12 cells with TriCurin. **a** Effect on p53. **b**–**d** Effect on HPV16 mRNAs: **b** 20862; **c** 20822; **d** 20850; W12 cells were treated with TriCurin at 8 μM for 6 or 24 h; extracts were prepared and analysed by RT-PCR analysis, as described in the “Methods” section. Fold change indicates relative expression in TriCurin-treated vs. control cells. For 24 vs. 0 h, the *p* values for p53 are all <0.01; for 24 vs. 6 h, the *p* values for p53 and E6 are <0.05; for 6 vs. 0 h, the *p* values for E1, E2, E4 and E6 are <0.01 (Supplementary Tables S3 and S4a–c).

To gain insight into the action of TriCurin, we examined the effect of TriCurin (90% curcuminoids) on p53 and HPV16 mRNAs in W12 cells, using RT-PCR. The results are displayed in Supplementary Table S3 and Fig. 2.

#### Effect of TriCurin on the expression of p53

RT-PCR indicated that TriCurin suppressed the expression of p53 at 6 h and activated the expression at 24 h in the three W12 cell lines, episomal, type 1 and 2 integrants. The relative order of activation of p53 expression at 24 h was 20862, 20822 and 20850, which relates to the state of integration of the HPV16 genome (episomal or integrated and the nature of the integration, type 1 or 2) (Fig. 2a). The differences between the relative levels of p53 mRNA at 0, 6 and 24 h in the three cell types are significant for 24 vs. 0 h or 6 h (*p* < 0.01); for 6 vs. 0 h, except for 20822 cells (*p* < 0.05) (Supplementary Table S4A–C).

It is of interest to monitor the effect of TriCurin on the mRNA levels arising from genes responsive to p53, such as p21 (cyclin-dependent kinase inhibitor 1A) and *MDM2* (MDM2 proto-oncogene). In preliminary experiments on W12 20822 (type 1 integrant) cells (the cells most sensitive to effects on HPV mRNA, by RT-PCR analysis), TriCurin activated the expression of p21 at 6 h (2.00-fold) and 24 h (2.04-fold); and the expression of MDM2 at 6 h (2.55-fold) and 24 h (1.2-fold) (data not shown). MDM2, which encodes a nuclear-localised E3 ubiquitin ligase, is the major negative regulator of p53. The effects on MDM2 are the inverse of the effects on p53. To explore if the biphasic effects of TriCurin on p53 could be related to effects on nuclear factor-κB (NF-κB), we examined the effect on the expression of NF-κB1, in type 1 20822 cells; TriCurin activated the expression of NF-κB1 at 6 h (1.68-fold) and 24 h (1.19-fold) (data not shown). The effects on NF-κB1 are the inverse of the effects on p53.

#### Effect of TriCurin on the level of HPV16 mRNAs

To evaluate the action of TriCurin, the effect of TriCurin on the expression of HPV16 early viral genes (*E1, E2, E4, E6* and *E7*) was assayed, using RT-PCR.

Concerning HPV16 mRNAs, the order of sensitivity of the cell lines was: (type 1) 20822 > (type 2) 20862 > (episomal) 20850 (Fig. 2b–d). The early genes *E1* and *E2* were the most sensitive as follows: (20822) E1 and E2 at 6 h; (20862) E2 at 6 h; (20850) E1 and E2 at 24 h. At 6 h, the relative reduction for E1 is: 20822, 0.07; 20862, 0.25; 20850, 0.46. The 20822 cells at 6 h are as sensitive as the 20850 cells at 24 h. For 20822 cells, at 6 h, the sensitivity decreases 6.25-fold from the early to the late genes. At 24 h, for 20850 cells, there is a 4.26-fold decrease in sensitivity from the early to the late genes; for 20862 cells, there is a 1.57-fold decrease. There is thus a greater effect on early genes in episomal and type I integrants, and on later genes in type II integrants. The differences between the relative level of HPV16 mRNAs at 0, 6 and 24 h in the three cell types are significant for 0 vs. 24 h (*p* < 0.01); for 6 vs. 24 h, for E6 (*p* < 0.05); for 0 vs. 6 h, except for E7 on 20850 cells (*p* < 0.01) (Supplementary Table S4A–C).

#### Comparison of the effects of TriCurin on mRNA expression and cell proliferation

The order of sensitivity of the cells is (IC_50_ value within parentheses): 20850 (2.7 μM); 20862 (6.25 μM); 20822 (8.25 μM) (data not shown). The lowest concentrations of TriCurin that induced significant effects on the three cell lines are: 2 μM on 20850 (*p* = 0.00650); 2 μM on 20822 (*p* = 0.0283); 2 μM on 20862 (*p* = 0.0549) cells (Supplementary Table S5A). TriCurin is more active on episomal cells vs. type 1 or type 2 integrant cells (the cells were incubated for 72 h, instead of 120 h, as in the earlier experiments), whereas the order of sensitivity of the cell lines by RT-PCR was: 20822, 20862 and 20850 cells. TriCurin is more active than tanshinone IIA on W12 episomal cells (IC_50_: 2.7 vs. 9.4 μM, respectively; data not shown), but the reverse is true for type 1 and 2 integrants (L.S. Einbond et al., unpublished results). The lowest concentrations of the two compounds that induced significant effects are: TriCurin (2 μM) (*p* = 0.00650); tanshinone IIA (4 μM) (*p* = 0.0196) (Supplementary Table S5B).

### The growth inhibitory effect of TriCurin in combination with phytochemicals

Since cancer alters multiple genes, we assayed the effects of combinations of TriCurin with active phytochemicals.

#### The effect of TriCurin containing turmeric (95% curcuminoids)

We first replaced turmeric (90% curcuminoids) in TriCurin with turmeric (95% curcuminoids) and found that turmeric (95% curcuminoids), as predicted, enhanced the activity. When the growth inhibitory activity of the two TriCurin preparations (95% and 90% curcuminoids) was assayed in the same experiment, the respective values were ~1.2 and 4.3 µM; the fold difference was ~3.5-fold.

#### Effect of combinations of TriCurin plus tanshinone IIA

Since TriCurin and tanshinone IIA appear to alter different pathways in the W12 cells, we assayed the effect of combinations of these phytochemicals.

##### Combination of TriCurin (90% curcuminoids) plus tanshinone IIA

The IC_50_ values for tanshinone IIA and TriCurin (90% curcuminoids) alone were ~13 and 6 μM on HeLa cervical cancer cells.

**Fig. 3:**
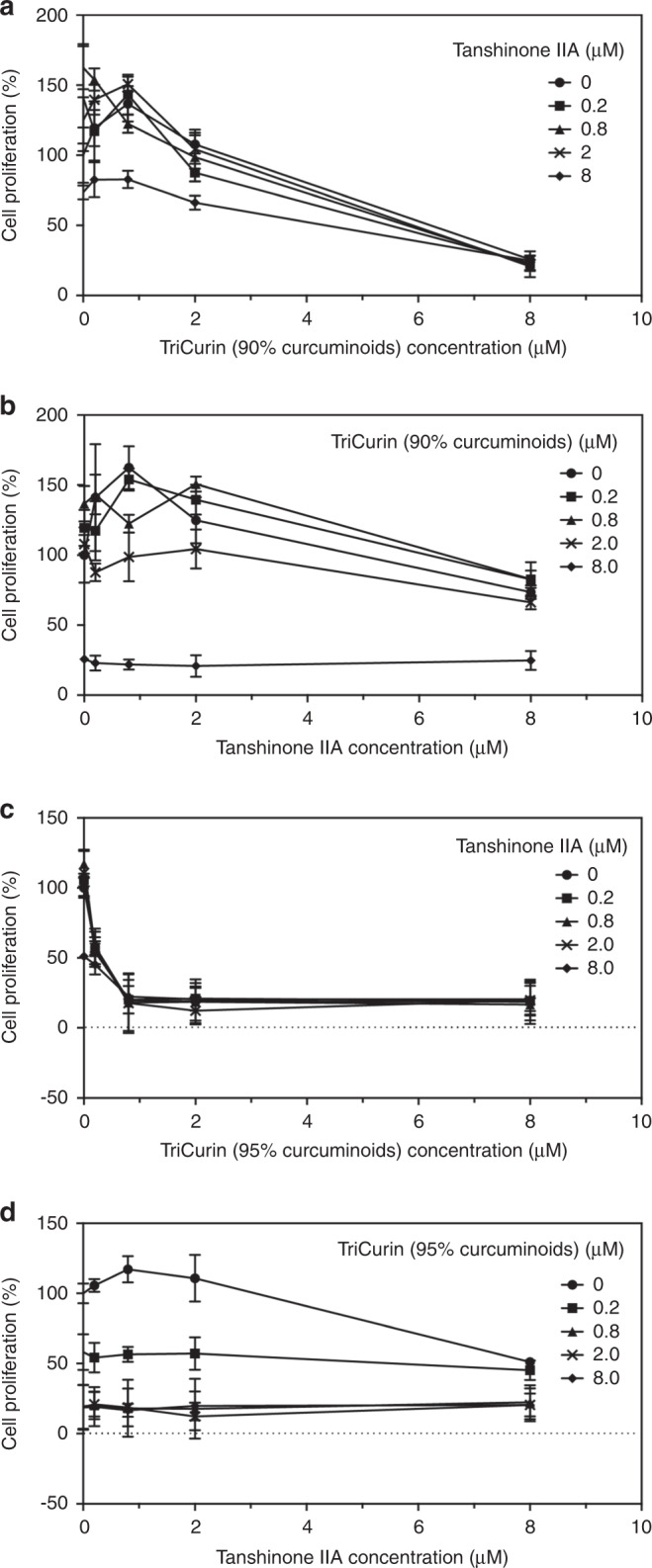
Growth inhibitory activity of combinations of phytochemicals: TriCurin (90 or 95% curcuminoids) plus tanshinone IIA on HeLa cells. **a**, **b** Combination of TriCurin (90% curcuminoids) plus tanshinone IIA. **a** *x*-axis: TriCurin (90% curcuminoids); **b** *x*-axis: tanshinone IIA; **c**, **d** combination of TriCurin (95% curcuminoids) plus tanshinone IIA; **c** *x*-axis: TriCurin (95% curcuminoids); **d** *x*-axis: tanshinone IIA. We treated HeLa cells with all combinations of four concentrations of each of the agents tested and a solvent control.^18^ Cells were exposed to increasing concentrations of agents for 96 h and the number of viable cells was determined by the EZQUANT assay.

When increasing concentrations of both TriCurin (90% curcuminoids) and tanshinone IIA were combined, at a dose of TriCurin 2.0 μΜ, the percent viable cells decreased from 107.84% with TriCurin alone to 87.64% with tanshinone IIA 0.2 μM, to 98.69% with tanshinone IIA 0.8 μΜ, to 104.29% with tanshinone IIA 2.0 μΜ, to 66.22% with tanshinone IIA 8 μΜ (*p* < 0.01) (Fig. 3a, b). Thus, tanshinone IIA enhances the growth inhibitory effect of TriCurin (90% curcuminoids) on the human cervical cancer cell line HeLa. The CI for the combination of tanshinone IIA (0.2 μΜ) and TriCurin (8 μΜ0 was ~0.88 (*p* < 0.001), indicating slight synergy.

##### Combination of TriCurin (95% curcuminoids) plus tanshinone IIA

The IC_50_ values for tanshinone IIA and TriCurin (95% curcuminoids) alone were ~8 and 0.2 μM, respectively, on HeLa cervical cancer cells. Increasing concentrations of tanshinone IIA were then combined with increasing concentrations of TriCurin (95% curcuminoids) (Fig. 3c, d). At a dose of TriCurin 0.2 μΜ, the percent viable cells decreased from 58.15% with TriCurin alone to 54.20% with tanshinone IIA 0.2 μΜ, to 56.55% with tanshinone IIA 0.8 μΜ, to 57.17% with tanshinone IIA 2.0 μΜ and to 45.24% with tanshinone IIA 8 μΜ (*p* < 0.01). Thus, tanshinone IIA enhances the growth inhibitory effect of TriCurin (95% curcuminoids) on the human cervical cancer cell line HeLa. The CI for the combination of TriCurin (95% curcuminoids) (0.8 μΜ) and tanshinone IIA (0.2 μΜ) was ~0.84 (*p* < 0.001), indicating slight synergy (Supplementary Table S6).

In a repeat experiment, the IC_50_ values for tanshinone IIA and TriCurin (95% curcuminoids) alone were ~6.7 and 3.5 μM, respectively, on HeLa cervical cancer cells. When increasing concentrations of both TriCurin (95% curcuminoids) and tanshinone IIA were combined, at a dose of TriCurin 1.6 μΜ, the percentage of viable cells increased from 92.22% with TriCurin alone to 94.88% with tanshinone IIA 0.2 μM, decreased to 36.10% with tanshinone IIA 0.8 μΜ, to 44.29% with tanshinone IIA 2.0 μΜ and to 19.46% with tanshinone IIA 8 μΜ (data not shown). Thus, tanshinone IIA enhances the growth inhibitory effect of TriCurin (95% curcuminoids) on the human cervical cancer cell line HeLa. The CI for the combination of tanshinone IIA (0.8 μM) and TriCurin (95% curcuminoids) (1.6 μΜ) was ~0.34, indicating strong synergy (percent viable cells: tanshinone IIA (0.8 μM), 81.73%; TriCurin (95% curcuminoids) (1.6 μΜ), 92.22%; combination, 36.10%).

### Growth inhibitory effect of resveratrol on breast cancer cells

**Fig. 4:**
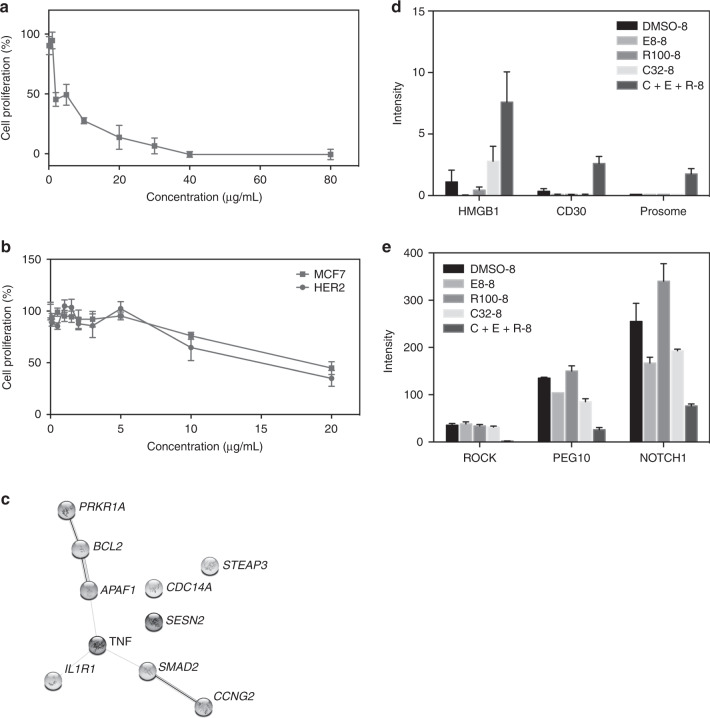
Growth inhibitory activity of resveratrol on breast cancer cells: MDA-MB-453, and MCF7 and MCF7(Her2); and gene expression analysis of the effects of TriCurin at 32 μM for 8 h on HeLa cells. **a**, **b** Breast cancer cells: **a** MDA-MB-453; **b** the genetically matched pair of cells MCF7 and MCF7(Her2); cells were exposed to increasing concentrations of agents for 96 h and the number of viable cells was determined by the MTT assay. **c**–**e** Gene expression analysis of the effects of TriCurin at 32 μM for 8 h on HeLa cells: **c** STRING (Functional Protein Association Networks: Search Tool for Retrieving INteracting Genes/proteins) analysis of the effects of TriCurin: significant p53, apoptosis and cell cycle genes (*p* < 0.05); up-regulated (fold > 10) and down-regulated (fold < −5) genes were subjected to STRING analysis. **d**, **e** Genes significantly altered by TriCurin compared to its individual compounds: **d** pro-apoptotic genes, CER vs. vehicle (*p* value): *CD3*0 (0.0059); *HMGB1* (0.0469); *Prosome* (0.0026); **e** anti-apoptotic genes, CER vs. vehicle (*p* value): *ROCK* (0.0018); *PEG10* (<0.0001); *NOTCH1* (0.0307); HeLa cells were treated with TriCurin (32 μM) and its individual compounds (curcumin: 32 μM; EGCG: 8 μM; resversatrol: 100 μM) and collected for RNA extraction at 8 h. Samples were prepared for gene expression analysis, as described in the “Methods” section. Data were extracted to show genes that were significantly affected by TriCurin compared to its individual compounds, curcumin (C), EGCG (E) or resveratrol (R). Some of the genes that showed the most pronounced response to TriCurin vs. the individual compounds.

To further evaluate the anti-cancer activity of the compounds, we assayed the growth inhibitory activity of resveratrol, since it is the most abundant component of TriCurin, on the oestrogen receptor (ER)-negative, Her2-overexpressing human breast cancer line MDA-MB-453; the IC_50_ value was: 1.91 μg/mL (8.32 μM) (Fig. 4a). The lowest concentration of resveratrol that induced significant effects on 453 cells is 0.1 μg/mL (0 vs. 0.1 μg/mL) (*p* = 0.0420) (Supplementary Table S7A). Thus, resveratrol strongly inhibits the growth of these cancer cells. In comparison, the chemotherapeutic agent cisplatin has an IC_50_ of 2.7 μg/mL (9.0 μM) in our assay (data not shown).

To lend insight into the role of Her2 in the action of resveratrol, we tested the effect of resveratrol on the genetically matched pair of cells MCF7 (ER-positive, Her2 low) and MCF7/Her2–18 (MCF7 cells are transfected with a full-length Her2 cDNA coding region, 45-fold increase Her2).^15^ The IC_50_ values were: MCF7, 18 μg/mL; 78 μM; MCF7(Her2), 15 μg/mL; 66 μM, indicating that sensitivity is slightly related to Her2 expression, but the Her2 pathway is not the major pathway (Fig. 4b). The lowest concentrations of resveratrol that induced significant effects on MCF7 and MCF7(Her2) cells are 10 μg/mL (*p* = 0.00120) and 0.1 μg/mL (*p* = 0.0130), respectively (Supplementary Table S7B).

### Transcriptomic effects of TriCurin on HeLa cells

In prior studies, we examined the effects of TriCurin on the protein levels in HeLa cells. In addition, HeLa cells were used to derive TriCurin’s synergistic molar ratio. Therefore, to better understand the mode of action of TriCurin, we examined the effect of TriCurin on HeLa cells, using microarray analysis. Cells were treated with 32 μΜ TriCurin or equal amounts of individual compounds (curcumin: 32 µM; EGCG: 8 µM; resversatrol: 100 µM) and collected for RNA extraction at 8 h.

#### Effects of TriCurin on p53, apoptosis and cell cycle pathway genes

Genome-wide expression analysis indicates that TriCurin alters the expression of p53, apoptosis and cell cycle pathway genes. The most highly altered genes, in these pathways, after treating with TriCurin, 32 µM for 8 h, were: (up) (fold > 10) *IL1R1*, *SMAD2* and *PRKARIA*. The functions of these genes are as follows: *IL1R1* (Interleukin-1 receptor type 1), a receptor for IL1A, IL1B and IL1RN, functions in interleukin-1-dependent activation of NF-κB, MAPK and other pathways; *SMAD2* (mothers against decapentaplegic homologue 2; receptor-regulated SMAD (R-SMAD)) is an intracellular signal transducer and transcriptional modulator activated by TGF-beta (transforming growth factor) and activin type 1 receptor kinases; it may act as a tumour suppressor in colorectal carcinoma; *PRKAR2A* (cAMP-dependent protein kinase type II-alpha regulatory subunit) binds to anchoring proteins, such as MAP2 kinase.

The top down-regulated genes are (fold < −5): *TNF* (tumour necrosis factor), *STEAP*, *BCL2*, *APAF-1 (*apoptosis-activating factor-1), *CCNG2* (cyclin-G2), *APAF1*, *SESN2* and *CDC14A*. The functions of these genes are: *TNF* is a cytokine that binds to TNFRSF1A/TNFR1 and TNFRSF1B/TNFBR. It is mainly secreted by macrophages and can induce cell death of certain tumour cell lines: *STEAP*, endosomal ferrireductase, functions in erythroid iron homeostasis required for efficient transferrin-dependent iron uptake in erythroid cells; *BCL2* (apoptosis regulator), represses apoptosis in various cell systems; inhibits caspase activity; binds to *APAF-1*. *CCNG2*, functions in growth regulation and in negative regulation of cell cycle progression; *APAF1*, oligomeric Apaf-1 functions in activation of pro-caspase-9 (Apaf-3), and thereby caspase-3 and apoptosis; *SESN2*, functions as an intracellular leucine sensor; prevents TORC1 signalling; *CDC14A*, dual specificity protein phosphatase.

To reveal altered protein connections, we subjected the results of gene expression analysis (significant top up- and down-regulated genes) to STRING (Functional Protein Association Networks: Search Tool for Retrieving Interacting Genes/proteins) analysis, as displayed in Fig. 4c. The proteins at the hub of the pathways are BCL2, APAF1, TNF and SMAD2.

Since TriCurin activated the expression of p53, it is of interest to examine the effect of TriCurin on the mRNA levels of genes responsive to p53. Gene expression analysis indicates that TriCurin activated the expression of LRDD, TSC2 and SIAH1 and repressed the expression of El24, PTEN, ZMAT3, SESN2, CCNG2 and STEAP3.

#### Comparison of the effects of TriCurin and its individual compounds

Data were extracted to show genes significantly affected by TriCurin compared to its individual compounds, Curcumin (C), EGCG (E) or Resveratrol (R). TriCurin activated the expression of apoptotic (*HMGB1*, *CD30*, *Prosome*, *ABL1*, *OBSCN*, *ARHGEF17*, *MAPT*, *CAPN10*, *Prune2*, *ID3*, *BMF*, *FASTK*, *IREIa*, *PPP2R5B*) and cell cycle (*MAD1L1*, *CDK10*) genes, and repressed the expression of anti-apoptotic (*ROCK*, *ADAM17*, *NOTCH1*, *PEG10*, *GRP94*, *SERBP1*, *USP47*, *P62*, *NK4A2*) genes. Some of the genes that showed the most pronounced response to TriCurin vs. the individual compounds are shown in Fig. 4d (apoptotic genes: (CER vs. vehicle (*p* value): *CD30* (0.0059); *HMGB1* (0.0469); *Prosome* (0.0026)) and Fig. 4e (anti-apoptotic genes: (*CER* vs. vehicle (*p* value): *ROCK* (0.0018); *PEG10* (< 0.0001); *NOTCH1* (0.0307)).

### Stability testing of TriCurin-Enpen cream

**Fig. 5:**
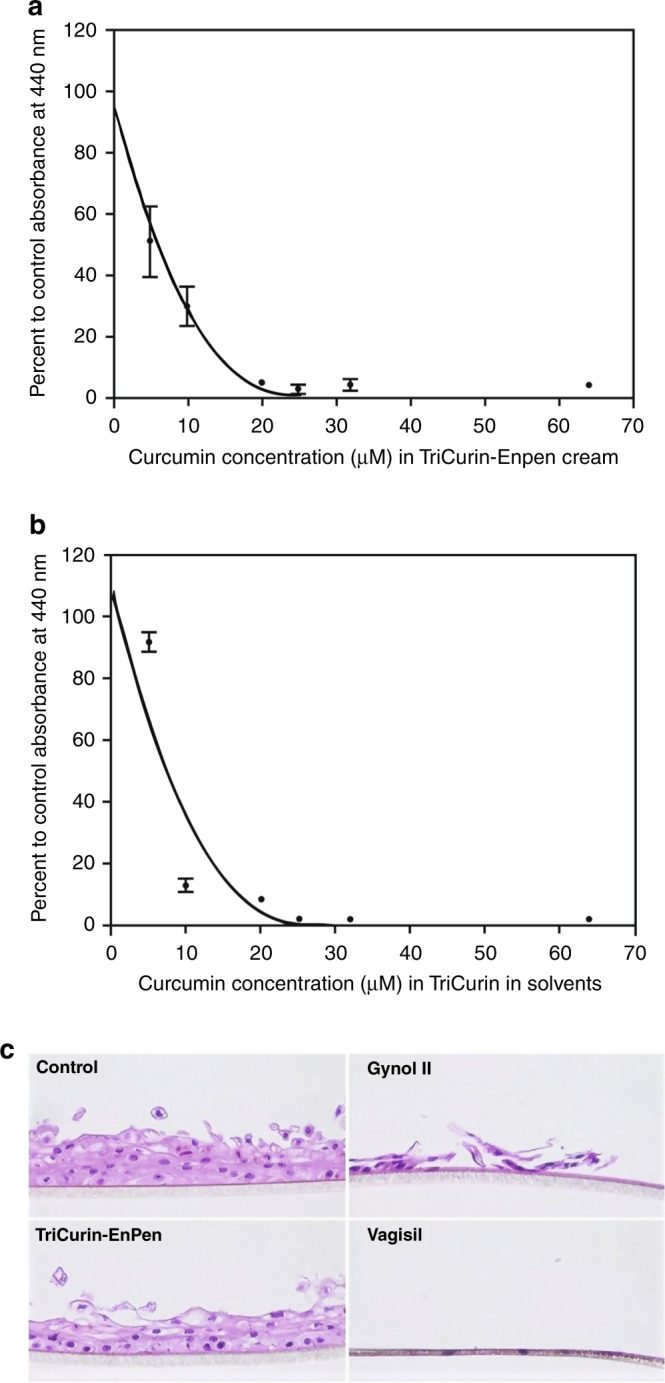
Analysis of curcumin (TriCurin) stability and toxicity; evaluation of the safety and toxicological profile: TriCurin and Enpen on human EpiVaginal tissues. **a**, **b** Biologic stability of TriCurin-Enpen cream: **a** TriCurin-Enpen; **b** TriCurin alone; HeLa cells were treated with TriCurin-Enpen cream or TriCurin alone (in solvents), at increasing concentrations and the percent survival determined, using tetrazolium assays, as described in the “Methods” section; IC_50_: 6 ± 1.9 µM for TriCurin in Enpen; IC_50_: 7 ± 0.5 µM for TriCurin only; **c** histology: TriCurin and Enpen on human EpiVaginal tissues; for histologic examination, entire EpiVaginal tissue implants were treated with either Enpen-TriCurin, Enpen (Control) or with two positive control creams Vagisil (benzocaine 5%, resorcinol 2%) and Gynol II (nonoxynol-9 (3%) for 4h and stained with haematoxylin and eosin (H & E) (magnification ×100), as described in the “Methods” section.

TriCurin-Enpen cream (20%) was made based on the maximum amount of dry compounds able to be emulsified, producing a homogeneous cream mixture confirmed by fluorescence microscopy (Supplementary Fig. S2). To evaluate if the microemulsion-based cream called EnPen (an amphoteric microemulsion) was able to keep the compounds active, a biologic assay was developed based on the activity against HeLa cells. The cytotoxicity of TriCurin-Enpen cream (IC_50_: 6 ± 1.9 µM; *N* = 3, an average of time 0, time 7 days and time 3.5 months) at time 0 is comparable to the cytotoxicity of TriCurin when dissolved in solvents (IC_50_: 7 ± 0.5 µM for TriCurin only; *N* = 6, for each concentration 6 wells were used), on HeLa cells, measured by a tetrazolium-based assay (Fig. 5a, b).^10^ The difference between the two values is not significant (*p* = 0.240) (Supplementary Table S8). TriCurin in Enpen still is stable after 3.5 months showing cytotoxicity against HeLa cells similar to time 0, by MTT assay, as shown in Table 1A. The percent viability at 4 µM is as follows: (0 time) (EnPen) 63%; (TriCurin-EnPen) 30%; (7 days) (EnPen) 62%; (TriCurin-EnPen) 51%; (3.5 months) (EnPen) 53%; (TriCurin-EnPen) 29% (TriCurin-Enpen vs. Enpen, *p* = 0.00483).

**Table 1:** Stability and toxicology study of Enpen and TriCurin-Enpen creams. (A) 3.5 months test for Tricurin-EnPen cream. (B) Toxicology study of EnPen: MTT assay % viability. (C) Toxicology study of TriCurin-EnPen: MTT assay % viability.

(A) 3.5 months Test for TriCurin-EnPen cream								
Concentration (µM)	EnPen alone			TriCurin-EnPen			*T* (EnPen-TriCurin-EnPen)*	
	Mean	SD	% of Viability	Mean	SD	% of Viability	*T* statistics	*P* value
0	1.672	0.108	100	1.665	0.218	100	0.076	0.940
1	1.333	0.279	80	1.172	0.312	70	1.018	0.326
2	1.031	0.336	62	0.880	0.310	52	0.874	0.397
4	0.892	0.240	53	0.478	0.223	29	3.343	0.005
8	0.722	0.182	43	0.313	0.105	19	5.15	0.000
16	0.665	0.200	40	0.120	0.033	7	7.113	0.000
32	0.588	0.151	35	0.021	0.010	1	9.913	0.000

(B) Toxicology study of EnPen: MTT assay % viability									
Results				Two-tailed *T* test statistics					
Time (h)	EnPen, mean ± SD	Gynol II, mean ± SD	1% Triton X-100, mean ± SD	EnPen vs. Gynol II		EnPen vs. 1% Triton X-100		Gynol II vs. 1% Triton X-100	
				T-s*	P-v**	T-s*	P-v**	T-s*	P-v**
0	100.00 ± 5.76	100.00 ± 5.76	100.00 ± 5.76	0.000	1.000	0.000	1.000	0.000	1.000
1	99.04 ± 7.66	78.13 ± 2.11	62.49 ± 17.10	3.722	0.020	2.759	0.051	1.284	0.269
4	105.00 ± 0.96	67.11 ± 6.69	4.71	7.928	0.001	73.871	0.000	6.595	0.022
24	98.06 ± 3.46	3.64 ± 1.03	1.87 ± 0.24	36.988	0.000	39.222	0.000	2.367	0.077

(C) Toxicology study of TriCurin-EnPen: MTT assay % viability						
Time (h)	M-EnPen, mean ± SD	TriCurin-EnPen, mean ± SD	Gynol II, mean ± SD	Vagisil (anti-itch cream), mean ± SD	*T* test (EnPen vs. Vagisil)	
					*T* statistics	*P* value
0	100.00 ± 3.02	100.00 ± 3.02	100.00 ± 3.02	100.00 ± 3.02	0.000	1.000
1	101.33 ± 1.20	N/A	75.51	102.60 ± 2.45	0.658	0.546
4	76.50 ± 4.20	N/A	25.48	50.72 ± 1.89	7.916	0.001
24	79.75 ± 2.30	67.49	3.38	2.48 ± 0.09	47.598	0.000

(A) Enpen and TriCurin-Enpen were stored in the dark at 4 °C for 3.5 months. HeLa cells were exposed to increasing concentrations of Enpen or TriCurin-Enpen for 96 h and the number of viable cells was determined by the MTT assay, as described in the “Methods” section.

*Two-tailed *T* test.

(B,C) Organotypic human vaginal-ectocervical (VEC) tissue (EpiVaginal, MaTtek, MA) was used to test the toxicity of:

(B) Enpen compared to two positive control creams known to be toxic: Gynol II, 1% Triton X-100, as described in the “Methods” section.

*T-s: *T* statistics; **P-v: *p* value.

(C) TrCurin-Enpen and Enpen cream compared to two positive control creams known to be toxic Vagisil (benzocaine 5%, resorcinol 2%) and Gynol II (nonoxynol-9 (3%), as described in the “Methods” section.

### Evaluation of the safety and toxicological profile: TriCurin and Enpen on human EpiVaginal tissues

The safety and toxicity of the compounds in TriCurin were determined using a 3D in vitro model (EpiVaginal tissue) generated from human donor VEC cells; the model is used by industry to test feminine hygiene products (Supplementary Fig. S3). The endpoints of interest are tissue viability and histology. The results are displayed in: (MTT assays: two separate experiments) Table 1B, C and (histology) Fig. 5c.

To evaluate if Enpen alone is non-toxic on human EpiVaginal tissues, EpiVaginal cervical tissues were treated with Enpen or with two positive control creams known to be toxic Gynol II (nonoxynol-9 (3%)) or Triton X-100. Treatment with Enpen resulted in 98% viability at 24 h, whereas treatment with Gynol II or 1% Triton X-100 yielded 3.64% (EnPen vs. Gynol II: *p* < 0.001) and 1.87% (Enpen vs. Triton X-100: *p* < 0.001) viability, respectively, at 24 h (Table 1B). Thus, Enpen is not toxic.

To evaluate if TriCurin-Enpen is non-toxic on human EpiVaginal tissues, EpiVaginal cervical tissues were treated with Enpen, Enpen-TriCurin or with two positive control creams known to be toxic Vagisil (benzocaine 5%, resorcinol 2%) and Gynol II (nonoxynol-9 (3%)). Treatment with Enpen resulted in 20.25% decrease in viability (EnPen vs. Vagisil: *p* < 0.001), while TriCurin resulted in 32.51% decrease in viability (Table 1C). According to MatTek, the manufacturer of EpiVaginal Tissue, any substance is considered toxic if cell death is 50% or more at 24 h. Thus, Enpen is not toxic and TriCurin-Enpen microemulsion appeared to also be non-toxic in preliminary testing, based on the EpiVaginal model.

To confirm the safety and toxicity of TriCurin-Enpen, EpiVaginal cervical tissues were treated with either control (negative control), TriCurin-Enpen, Gynol II or Vagisil for 4 h and examined using histology. TriCurin-Enpen appears to be non-toxic in comparison to Gynol II and Vagisil (Fig. 5c).

## Discussion

To our knowledge, we are the first to show that the phytochemicals in the TriCurin mixture inhibit the growth of various HPV (+) cells, including W12 cells derived from a pre-cancerous cervical lesion, harbouring extrachromosomal or integrated HPV16 DNA. Moreover, the synergistic mixture of phytochemicals when formulated as TriCurin inhibits viral transcripts and alters key cellular genes used by HPV to transform keratinocytes, especially the p53 pathway. This preclinical efficacy data support the possibility that the TriCurin mixtures may target the spectrum of epithelial diseases HPV induces.

It is important that previous studies show that TriCurin is selective for malignant vs. non-malignant cells,^10^ indicating that this agent possesses specificity and may have limited toxicity in patients, especially if delivered locally. The present studies confirm that TriCurin has selective cytotoxicity for HPV (+) W12 cells and cancer cell lines. Our preliminary data using donor VEC cells in the 3D tissue implants show no toxicity on H&E examination after TriCurin-Enpen is administered topically.

In addition, we showed that TriCurin displays potent growth inhibitory activity on W12 cells. The order of activity of the TriCurin mixture and phytochemicals is: TriCurin > EGCG > resveratrol or curcumin. TriCurin and EGCG possess comparable activity on 20850 cells containing episomal HPV DNA; curcumin and resveratrol are less active. TriCurin and EGCG alone may, in particular, be useful to prevent the development of cervical cancer. However, EGCG rapidly degrades in aqueous media.

It is interesting to note that resveratrol strongly inhibits the growth of Her2-overexpressing MDA-MB-453 cells. To gain insight into the role of Her2 in the action of resveratrol, we tested the effect of resveratrol on the genetically matched pair of cells MCF7 and MCF7(Her2) and found that the activity partially, but not strongly, correlates with Her2 expression.

In prior studies,^10^ we found that, in HeLa cells, TriCurin activates the expression of p53, while suppressing the expression of NF-κB, in HPV16 (+) TC-1 cells. HeLa cells contain integrated and amplified HPV18 DNA missing 2–3 kb sequences from the E2 to the L2 region.^21^ Further, in mouse TC-1 epithelial cells that express c-Ha-ras and HPV16 E6 and E7, TriCurin and curcumin increase the level of p53, acetyl-p53, the activated form of p53, and active caspase-3. It is important that TriCurin is several folds more active than curcumin: 4.7-fold for E6 reduction and 6-fold for acetyl-p53 induction.

Our present studies confirm that in W12 cells, TriCurin activates the expression of p53 and represses the expression of HPV16 viral genes *E6* and *E7* at 24 h. In addition, we found that TriCurin represses the expression of p53 at 6 h and of HPV16 *E1*, *E2* and *E4* at 6 and 24 h in W12 cells. It is important to highlight that TriCurin repressed the expression of *E1* and *E2*. Both these genes have been difficult to target pharmacologically, yet are essential for viral replication.^22^

In regards to p53, TriCurin slightly reduced the expression at 6 h and activated the expression at 24 h in the three W12 cell types. The order of activation was type 2 integrant (20862), type 1 integrant (20822) and episomal (20850), which relates to the state of integration.

The biphasic effect on p53 may relate to an effect on NF-κB. We have observed a biphasic effect on growth, mRNA and protein levels after treatment with various phytochemicals. Actein and an extract of black cohosh^17,23^ induce two phases of the integrated stress response depending on the dose and duration of treatment; they first stimulate the expression of NF-κB and the survival phase, and second, repress the expression, leading to cell death. The biphasic effect of actein may relate to effects on calcium metabolism. Calcium regulates all IP3 receptors in a biphasic pattern.^23^ Modest increases enhance responses to IP3, whereas higher doses inhibit the response. An increase in intracellular calcium may lead to an increase in NF-κB activity. Actein induces a transient release of calcium; this release may cause a transient increase in NF-κB activity, which may result in anti-inflammatory activity, as shown for *S*-[6]-gingerol.^24^ TriCurin may induce a similar pattern of effects on NF-κB and the integrated stress response. Preliminary experiments indicate that TriCurin induced a biphasic effect on the expression of NF-κB1 in W12 type 1 integrant cells. In future experiments, we will assay the effect on NF-κB protein levels.

The present studies indicate that p53 is differently reduced or induced in the three W12 cell types. This finding may also relate to an effect on NF-κB. Curcumin has been shown to inactivate the transcription factors AP-1 and NF-κB, which may induce transcription of HPV *E6* and *E7*. The inhibition of NF-κB and resulting suppression of E6 result in induction of p53.^10^ The magnitude of the activation of p53 may relate to the magnitude of inhibition of NF-κB.

Concerning an effect on HPV16 mRNAs, the order of sensitivity of the cell lines was: (type 1) 20822 and (type 2) 20862 integrated clones, then (episomal) 20850 cells. The early genes *E1* and *E2* were the most sensitive as follows: (20822) E1 and E2 at 6 h; (20862) E2 at 6 h; (20850) E1 and E2 at 24 h. The repression was greater on early genes in episomal and type 1 integrants; and on later genes in type 2 integrants. TriCurin could exert a direct effect on viral gene expression or an indirect effect. One possibility is that TriCurin could alter the HDAC pathway, which could suppress the expression of HPV16 E6 and E7.^25^

In future experiments, we will confirm the effect of TriCurin on the level of important proteins, NF-κB, p53, acetyl-p53, cleaved-caspase-3, poly (ADP-ribose) polymerase and HPV16 E6, and on the mRNA levels of genes responsive to p53, at 6 and 24 h in W12 cells. In addition, we will examine the possibility that TriCurin may stabilise and extend p53 half-life.

The relative order of sensitivity of the cells as measured by PCR is: 20822, 20862 and 20850. The relative order of sensitivity by MTT assay is the inverse: 20850, 20862 and 20822. Thus, the effect on mRNAs cannot account for the growth inhibitory effect; other factors may contribute to the growth inhibitory effect observed. It is notable that for this experiment, TriCurin is more active than tanshinone IIA on 20850 (episomal) cells, whereas the inverse is true on type 1 and 2 integrant cells. This suggests that TriCurin may be especially effective to prevent the development of cervical cancer.

We passaged the 20850 cells two more times than the 20822 and 20862 cells to generate adequate cells for the RT-PCR experiments. To gain insight into the state of the DNA (episomal or integrated) in the W12 cells, we assessed the level of p53 and the HPV16 mRNAs at 0 time. For the replication genes *E1* and *E2*, the level of E1 in 20850 cells was 10 or more times the level in 20822 and 20862 cells. This finding is consistent with an episomal state for the viral genome. The level of all the viral mRNAs tested was reduced in 20822 and 20862 cells vs. 20850 cells, except for E6 in 20862 cells. These findings are consistent with earlier findings on the level of E6 proteins in 20850 and 20862 cells^26^ and are consistent with an episomal state for the viral genome in the 20850 cells. However, it is possible that the DNA integrated in some percent of the cells. In future experiments, we will examine 20850 cells, using Southern blot analysis, to further confirm the effects on extrachromosomal and integrated forms of HPV16 DNA.

To better understand the mode of action of TriCurin, we examined the effect of TriCurin and its individual phytochemicals on HeLa cells, using gene expression analysis. TriCurin activated the expression of apoptotic, p53 pathway and tumour suppressor genes and repressed the expressions of anti-apoptotic genes.

To reveal connections among the altered pathways, we subjected the results of gene expression analysis (significant up- and down-regulated p53 pathway, apoptosis and cell cycle genes to STRING (Functional Protein Association Networks) analysis (Fig. 4c). The genes at the hub of the pathways are *BCL2*, *APAF1*, *TNF* and *SMAD2*. The top up-regulated genes alter the NF-κB and TGF pathways and function as tumour suppressor genes; the top down-regulated genes repress apoptosis and prevent Torc2 signalling and play a role in gene regulation. In addition, gene expression analysis indicates that TriCurin altered the expression of genes responsive to p53: (up) *LRDD*, *TSC2* and *SIAH1* and (down) *El24*, *PTEN*, *ZMAT3*, *SESN2*, *CCNG2* and *STEAP3*.

TriCurin inhibits cell proliferation, yet reduces the expression of PTEN at 8 h. A possible explanation for these findings is that TriCurin may induce a transient increase in PTEN and a stimulation of cell proliferation, which may be related to the effects we have observed for NF-κB1 (increase) and p53 (decrease) at early times (6 h). The action of TriCurin may resemble that of the phytochemicals black cohosh and actein, which induce two phases of the integrated stress response (iSR), a transient stimulation of proliferation and NF-κB activity, followed by a decrease.^17,23^ Concerning p53 and PTEN, Stambolic et al.^27^ have shown that p53, via PTEN, functions in negative regulation of cell survival. In addition, the study of Feng et al.^28^ indicates that miR-130a induces cervical cancer cell proliferation by targeting PTEN, dependent on NF-κB. It will be important to assess the effects of TriCurin, at early and later times, 6 and 24 h, on cell proliferation, NF-κB, miR-130a and PTEN.

Further, TriCurin activated the expression of apoptotic genes and repressed the expression of anti-apoptotic genes more than its individual phytochemicals. The genes that showed the most pronounced response to TriCurin vs. its individual compounds are shown in Fig. 4d (apoptotic genes: *HMGB1*, *CD30*, *Prosome*) and Fig. 4e (anti-apoptotic genes: *ROCK*, *ADAM17*, *NOTCH1*).

Since cancer has been associated with mutations in multiple genes, we assessed the growth inhibitory effects of combinations of active phytochemicals. When we substituted turmeric containing 95% curcuminoids, for turmeric containing 90% curcuminoids, the activity of TriCurin increased >3.5-fold.

Tanshinone IIA enhanced the growth inhibitory effect of TriCurin containing 90% and 95% curcuminoids. Tanshinone IIA synergised with TriCurin (turmeric, 95% curcuminoids). In addition, in a repeat assay, the IC_50_ of TriCurin was 3.55 µM and the combination with tanshinone IIa (0.8 µM) was 1.2 µM. Thus, the combination of TriCurin (95% curcuminoids) and tanshinone IIA may be useful for HPV (+) cancer therapy. The use of drug combinations may allow the use of lower doses, which may potentially lessen toxic side effects. These botanicals are attractive since, for the most part, they have a history of safe use and are inexpensive,

Concerning in vivo activity, we previously showed that intralesional injection of TriCurin into tumours formed after HPV16 (+) TC-1 cells were implanted subcutaneously resulted in 80–90% decrease in tumour size. Further, TriCurin injected subcutaneously in tumour-naive mice yielded no adverse effects.^10^

A recent study found that when TriCurin is injected into mice bearing tumour xenografts from a tumorigenic cell line expressing HPV16 E6 and E7 oncoproteins, it suppresses the growth of the tumours at concentrations that are not adequate to kill tumour cells in vitro.^29^ The authors argue that TriCurin activates an innate and adaptive immune response that drives immune clearance.

Specifically, injection of TriCurin had two effects: it repolarised tumour-associated macrophages from the M2-like form, which promotes tumour growth, to the M1-like phenotype, which produces an anti-tumour phenotype. In addition, TriCurin affected IL-12 signalling to recruit activated NK cells and cytotoxic T-lymphocytes (CTLs) to the tumour. It will be worthwhile to determine if the substitution of turmeric containing 95% curcuminoids, instead of 90%, in TriCurin, or the combination of TriCurin with tanshinone IIA enhances immune response.

The use of curcumin in clinical trials has shown limited utility due to its rapid degradation in an aqueous environment, poor systemic bioavailability and overall low target tissue delivery. Compounds with antioxidant activity that also developed intermolecular interactions with curcumin, leading to close π–π stacking of aromatic molecules in an aqueous environment, could stabilise curcumin. Our previous studies indicate that EGCG and resveratrol stabilise curcumin in aqueous media.^10^ This finding suggests that our combinatorial approach can overcome many of curcumin’s limitations seen in prior clinical trials. To advance towards clinical testing, a microemulsion-based cream was developed to specifically deliver TriCurin locally. Our findings indicate that TriCurin in a microemulsion is stable and non-toxic. The major advantage of topical drugs is that significant tissue concentrations can be achieved while reducing any systemic side effects. HPV initially infects basal epithelial cells, creating a reservoir in keratinocyte stem cells along the basement membrane of epithelial tissue. An effective therapeutic will need to reach these basal cells to eradicate HPV. Our prior studies show topical application of TriCurin dispersed in a cream (a uniform mixture of 20% TriCurin in a topical cream base VanPen (TriCurin-VanPen)) resulted in complete permeation of curcumin (fluorescent) across the full thickness of the skin at 20 h.^10^ Further, when TriCurin was injected subcutaneously in mice not bearing tumours, no adverse effects were observed. TriCurin is, therefore, a safe and promising therapy against HPV-induced diseases.

## Conclusions

Our findings show that TriCurin is effective against a variety of HPV (+) cells, including the unique cell line W12, derived from a cervical pre-cancerous lesion containing cells with extrachromosomal or integrated HPV16 DNA. The growth of three W12 cell clones, episomal, type 1 and 2 integrants can be inhibited and the relative order of activity is TriCurin, EGCG, curcumin or resveratrol. RT-PCR indicates that TriCurin activates the expression of p53 and reduces the expression of HPV16 mRNAs E1, E2, E4, E6 and E7 at 24 h in the three W12 cell clones. Genome-wide expression analysis shows that TriCurin activates the expression of pro-apoptotic genes and represses the expression of anti-apoptotic genes, more than its individual compounds, in HeLa cells. The combination of tanshinone IIA with TriCurin (95% curcuminoids) exhibits additional synergy on HeLa cells. An organotypic tissue culture model shows that TriCurin microemulsion is stable and non-toxic. The phytochemical mixture TriCurin in the microemulsion-based cream is a promising therapeutic candidate for the prevention and treatment of HPV-induced cancers.

## Supplementary information

Supplementary Information

## Data Availability

The datasets used and/or analysed during the current study are available from the corresponding author on reasonable request. Supplementary information is available at the *British Journal of Cancer*’s website.
