# Supplementary material for: A novel cancer preventative botanical mixture, TriCurin, inhibits viral transcripts and the growth of W12 cervical cells harbouring extrachromosomal or integrated HPV16 DNA
Source: Br J Cancer. 2020 Dec 1;124(5):901–13. doi: 10.1038/s41416-020-01170-3 (PMC7921087; doi:10.1038/s41416-020-01170-3)
Supplement: Supplementary file 1 — Supplementary Information [file 41416_2020_1170_MOESM1_ESM.pdf]

Supplementary Information

Title: A novel cancer preventative botanical mixture, TriCurin, inhibits viral transcripts and the growth of W12 cervical cells harboring extrachromo-somal or integrated HPV16 DNA

Supplementary Information Figures

Figure S1. Structures of curcumin, EGCG and resveratrol and HPLC of turmeric (95% curcuminoids)

A. Curcumin

B. EGCG

C. Resveratrol

D. HPLC of turmeric (95% curcuminoids)

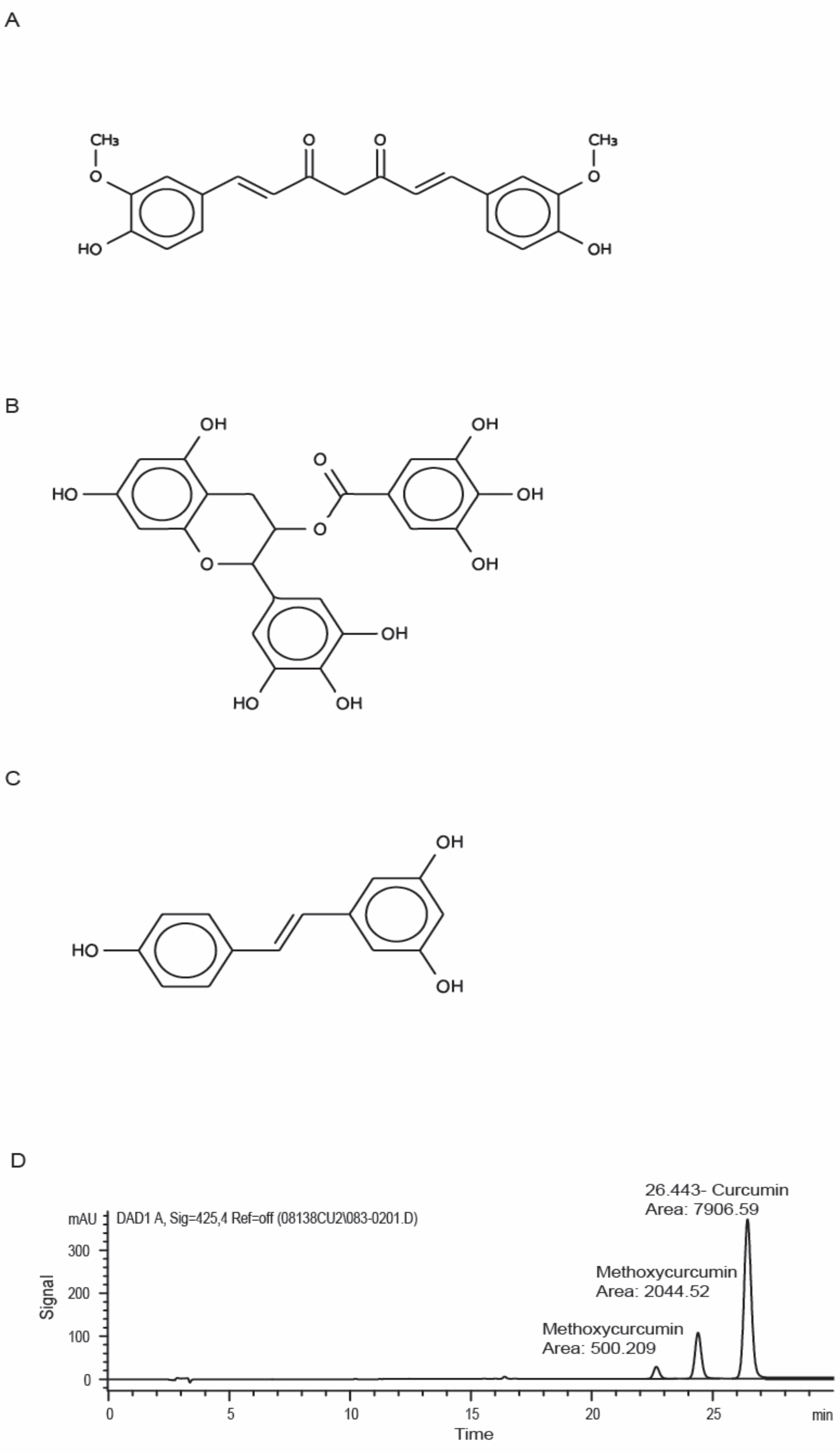
A
B
C
D
26.443- Curcumin
Area: 7906.59
Methoxycurcumin
Area: 2044.52
Methoxycurcumin
Area: 500.209
mAU
DAD1 A, Sig=425,4 Ref=off (08138CU2\083-0201.D)
Signal
Time
min

Figure S2. Identify maximum percentage of compounds that can be dissolved in the novel base called EnPen (an amphoteric nanoemulsion)

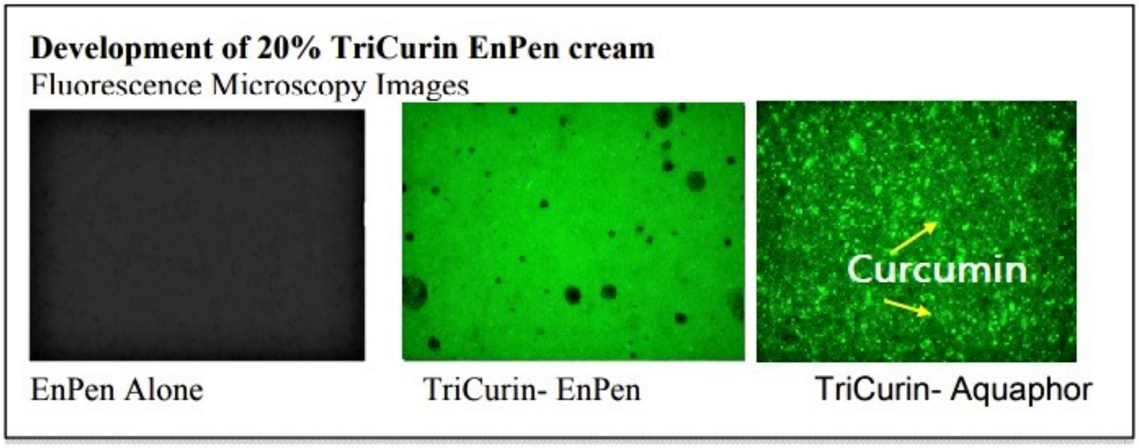

A. EnPen is the novel delivery emulsifying cream
   Has no green auto fluorescence
B. EnPen is the novel delivery vehicle for TriCurin
   Dissolves curcumin well (green)
C. Aquaphor is a commercially cream (used as a control vehicle)
   Curcumin remains undissolved (green particles)

20% Tricurin-EnPen cream was made based on maximum of dry compounds able to be emulsified producing homogenous curcumin auto-fluorescence

Figure S3. 3D Cervical Tissue Testing
EpiVaginal Assay

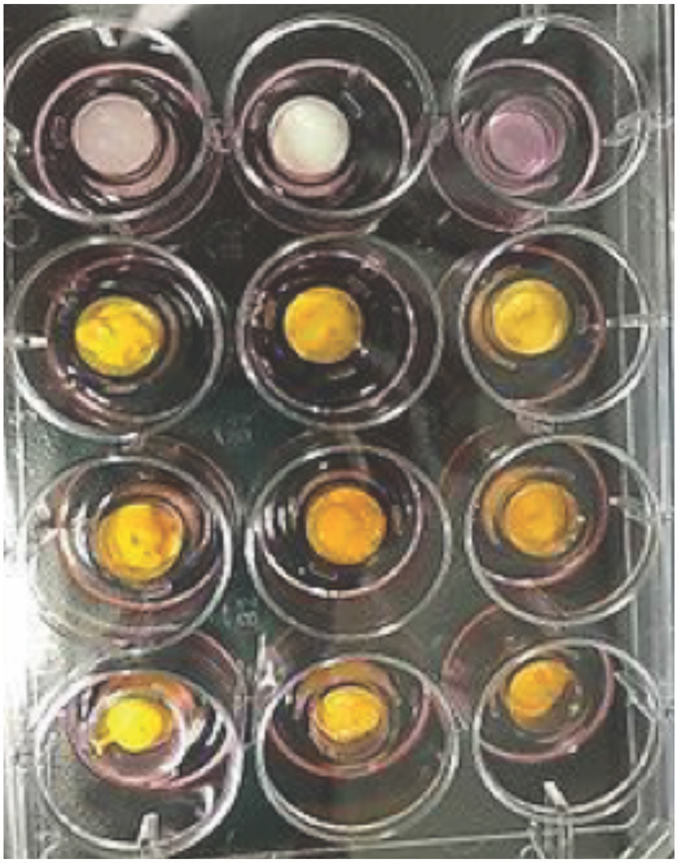

Control

TriCurin- EnPen

Curcumin-EnPen

TriCurin- Aquaphor

Illustration of EpiVaginal Assay

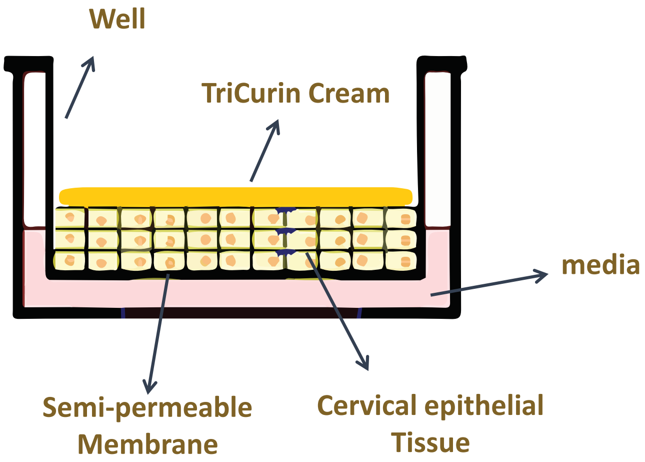

Supplementary Information Tables

Table S1. Primer sequences used in RT-PCR.

Gene name	Primer direction	Sequence	Reference
**TP53**	Forward	CACATGACGGAGGTTGTGAG	*
	Reverse	ACACGCAAATTTCCTTCCAC	
**E1**	Forward	AACGTGTTGCGATTGGTGTA	[S1]
	Reverse	TACGCAATTTTGGAGGCTCT	
**E2**	Forward	TGGAAGTGCAGTTTGATGGA	[S1]
	Reverse	CCGCATGAACTTCCCATACT	
**E4**	Forward	GACTATCCAGCGACCAAGATCAG	[S2]
	Reverse	CTGAGTCTCTGTGCAACAACTTAGTG	
**E6**	Forward	CTGCAATGTTTCAGGACCCA	[14]
	Reverse	TCATGTATAGTTGTTTGCAGCTCTGT	
**E7**	Forward	AAGTGTGACTCTACGCTTCGGTT	[14]
	Reverse	GCCCATTAACAGGTCTTCCAAA	
**P21**	Forward	TGGAGACTCTCAGGGTCGAAA	[S3]
	Reverse	GGCGTTTGGAGTGGTAGAAATC	
**NF-kB1**	Forward	CAGGAGACGTGAAGATGCTG	IDT**
	Reverse	AGTTGAGAATGAAGGTGGATGA	
**MDM2**	Forward	CAGGAGACGTGAAGATGCTG	IDT**
	Reverse	AGTTGAGAATGAAGGTGGATGA	
**GAPDH**	Forward	CAGCCTCAAGATCATCAGCA	*
	Reverse	GTCTTCTGGGTGGCAGTGAT	

*The mRNA sequences were obtained from the public GeneBank database (www.ncbi.nlm.nih.gov), and the primers were designed using Primer 3 software obtained from The Massachusetts Institute of Technology (frodo.wi.mit.edu/cgi-bin/primer3/primer3_www.cgi).
** The primer sequences were designed by Integrated DNA Technologies, Inc. (IDT).

Table S2. Relative level of Hpv16 and p53 mRNAs in the 3 cell types at 0 h

	Episomal DNA	Integrant: Type 1	Type 2
**Cells**	**20850**	**20822**	**20862**
**mRNA**			
**GAPDH**	1.00 +/- 0.11	1.00 +/- 0.12	1.00 +/- 0.03
**E1**	1.00 +/- 0.08	0.08 +/- 0.01	0.19 +/- 0.01
**E2**	1.00 +/- 0.03	0.76 +/- 0.18	0.44+/- 0.01
**E4**	1.00 +/- 0.07	0.41 +/- 0.05	0.83+/- 0.05
**E6**	1.00 +/- 0.05	0.62 +/- 0.02	1.25+/- 0.01
**E7**	1.00 +/- 0.04	0.64 +/- 0.05	0.85+/- 0.04
**p53**	1.00 +/- 0.07	0.18 +/- 0.02	0.38+/- 0.02

Table S2. Extracts were prepared and analyzed by Real-time RT-PCR, as described in Materials and Methods. Fold change indicates: relative expression in 20822 or 20862 cells versus 20850 cells.

Table S3. RT-PCR analysis of the relative level of HPV16 and p53 mRNAs in W12 cells, Tricurin treatment at 8 μM

	**Episomal DNA**			**Integrant: Type 1**		**Type 2**	
**Cells**	**20850**			**20822**		**20862**	
**Time (h)**	**0**	**6**	**24**	**6**	**24**	**6**	**24**
**mRNA**							
**GAPDH**	1	1	1	1	1	1	1
**E1**	1	0.46	0.047	0.07	0.05	0.25	0.07
**E2**	1	0.52	0.07	0.04	0.05	0.18	0.08
**E4**	1	0.41	0.14	0.2	0.04	0.26	0.07
**E6**	1	0.55	0.17	0.27	0.04	0.26	0.09
**E7**	1	0.51	0.2	0.25	0.05	0.25	0.11
**p53**	1	0.85	1.35	0.75*	1.7*	0.84	2

* repeat experiment

Table S3. W12 cells were treated with TriCurin at 8 μM for 6 or 24 hours; extracts were prepared and analyzed by Real-time RT-PCR, as described in Materials and Methods. Fold change indicates relative expression in TriCurin treated versus control cells (Figure 3).

Statistical Analysis of the Figures

In the interest of space, we have included portions of the statistical analyses of some figures, as examples, and can provide complete analyses upon request.
Non-significant: yellow

Table S4. Statistical analysis for Figure 2 (Table S3):

RT-PCR analysis of RNA obtained after treating W12 cells with TriCurin;

The p values for the PCR of p53 and 24h vs 0h are all <0.01; for 24h vs 6h, the p values are <0.05 for p53 and E6; for 6h vs 0h, the p values are <0.01 for E1, E2, E4 and E6.

Effects on p53 and HPV16 mRNAs:
A) 20850; B) 20822; C) 20862;

A) 20850

P-value of t-test(2-tails)			
	T-test(0h-6h)	T-test(0h-24h)	T-test(6h-24h)
**GAPDH**	1.000	1.000	1.000
**HPV-E1**	0.001	0.000	0.000
**HPV-E2**	0.000	0.000	0.000
**HPV-E4**	0.000	0.000	0.000
**HEADNECK-E2**	0.002	0.000	0.537
**HPV-E6**	0.001	0.000	0.000
**HPV-E7**	0.072	0.009	0.075
**p53**	0.032	0.004	0.000
Non-significant : Yellow highlighted			

B) 20822

P-value of t-test(2-tails)			
	**T-test(0h-6h)**	**T-test(0h-24h)**	**T-test(6h-24h)**
**GAPDH**	1.000	1.000	1.000
**HPV-E1**	0.000	0.000	0.296
**HPV-E2**	0.000	0.000	0.518
**HPV-E4**	0.001	0.000	0.000
**HPV-E6**	0.000	0.000	0.000
**HPV-E7**	0.000	0.000	0.000
**p53**	0.063	0.000	0.001
Non-significant : Yellow highlighted			

C) 20862

P-value of t-test(2-tails)			
	**T-test(0h-6h)**	**T-test(0h-24h)**	**T-test(6h-24h)**
**GAPDH**	1.000	1.000	1.000
**HPV-E1**	0.001	0.000	0.063
**HPV-E2**	0.001	0.003	0.584
**HPV-E4**	0.000	0.001	0.063
**HPV-E6**	0.000	0.000	0.013
**HPV-E7**	0.000	0.000	0.027
**p53**	0.046	0.000	0.000
Non-significant : Yellow highlighted			

Table S5. Statistical analysis for a comparison of the effects of TriCurin and tanshinone IIA on W12 cells;

A,B) Effect of Tricurin and tanshinone IIA on W12 cells used for RT PCR experiments: A) TriCurin on W12 cells; B) Tricurin and tanshinone IIA on 20850 cells

A) Effect of Tricurin on W12 cells used for RT PCR experiments:

Fig 4C	20822		20850		20862	
**T-test**	**t-statistic**	**p-value**	**t-statistic**	**p-value**	**t-statistic**	**p-value**
**0 vs 1**	1.660	0.148	1.566	0.168	1.563	0.169
**0 vs 2**	2.874	0.028	4.080	0.007	2.379	0.055
**0 vs 4**	9.594	0.000	15.311	0.000	7.356	0.000
**0 vs 8**	21.081	0.000	10.789	0.002	10.794	0.000

B) Effect of Tricurin and tanshinone IIA on 20850 cells used for RT PCR experiments:

**0 vs 1/0.8/0.004**	1.566	0.168	0.419	0.690	0.615	0.561
**0 vs 2/2/0.02**	4.080	0.007	0.996	0.358	0.153	0.883
**0 vs 4/4/0.1**	15.311	0.000	3.159	0.020	2.002	0.092
**0 vs 8/10/0.2**	10.789	0.002	7.864	0.000	1.562	0.169

Table S6. Statistical analysis for Figure 3 C,D.

Growth inhibitory activity of combinations of phytochemicals: TriCurin (95% curcuminoids) plus tanshinone IIA on HeLa cells;

Combination of TriCurin (95% curcuminoids) plus tanshinone IIA: Statistical analysis;

	**Mean1**	**Mean2**	**Mean3**	**Mean4**	**Mean5**
**Tashinone IIA**	0	0.2	0.8	2	8

**ANOVA based T-test**										
	**T(Mean 1-Mean2)**	**T(Mean 1-Mean3)**	**T(Mean 1-Mean4)**	**T(Mean 1-Mean5)**	**T(Mean 2-Mean3)**	**T(Mean 2-Mean4)**	**T(Mean 2-Mean5)**	**T(Mean 3-Mean4)**	**T(Mean 3-Mean5)**	**T(Mean 4-Mean5)**
**0**	9.244	17.759	17.862	17.974	8.515	8.617	8.730	0.103	0.215	0.113
**0.2**	11.379	18.900	18.718	19.100	7.521	7.339	7.721	0.181	0.201	0.382
**0.8**	13.381	21.872	21.754	22.195	8.490	8.373	8.813	0.118	0.323	0.441
**2**	11.849	20.550	21.759	20.099	8.701	9.910	8.250	1.209	0.450	1.660
**8**	1.283	6.341	6.752	6.757	5.058	5.469	5.474	0.411	0.416	0.005
**P value of ANOVA based T-test (2 tails)**										
**0**	0.000	0.000	0.000	0.000	0.000	0.000	0.000	0.918	0.830	0.911
**0.2**	0.000	0.000	0.000	0.000	0.000	0.000	0.000	0.857	0.841	0.704
**0.8**	0.000	0.000	0.000	0.000	0.000	0.000	0.000	0.907	0.747	0.661
**2**	0.000	0.000	0.000	0.000	0.000	0.000	0.000	0.230	0.654	0.101
**8**	0.204	0.000	0.000	0.000	0.000	0.000	0.000	0.682	0.678	0.996

Table S7. Statistical analysis for Figure 4A,B;

Growth inhibitory activity of resveratrol on breast cancer cells: MDA-MB-453, and MCF7 and MCF7(Her2);
A, B) breast cancer cells: A) MDA-MB-453; B) the genetically matched pair of cells MCF7 and MCF7(Her2);

A) MDA-MB-453

Simple t-test	
	**p-value**
DMSO vs 0.1	0.042
DMSO vs 1	0.251
DMSO vs 2	0.000
DMSO vs 5	0.000
DMSO vs 10	0.000
DMSO vs 20	0.000
DMSO vs 30	0.000
DMSO vs 40	0.000
DMSO vs 80	0.000

B) the genetically matched pair of cells
MCF7 and MCF7(Her2);

**Simple t-test**		**MCF7**		**MCF7(HER2)**	
		**T-statistic**	**P-value**	**T-statistic**	**P-value**
T(mean(0)-mean(0.1)	0 vs 0.1	2.181	0.052	2.958	0.013
T(mean(0)-mean(0.5)	0 vs 0.5	0.308	0.766		0.001
T(mean(0)-mean(1)	0 vs 1		0.187		0.241
T(mean(0)-mean(1.5)	0 vs 1.5		0.130		0.430
T(mean(0)-mean(2)	0 vs 2		0.128		0.006
T(mean(0)-mean(3)	0 vs 3		0.103		0.019
T(mean(0)-mean(5)	0 vs 5		0.124		0.561
T(mean(0)-mean(10)	0 vs 10		0.001		0.000
T(mean(0)-mean(20)	0 vs 20		0.000		0.000

Table S8. Statistical analysis for Figure 5 A,B.

Biologic stability of TriCurin-Enpen cream: TriCurin-Enpen vs TriCurin alone;

mean	SD	n
TriCurin-Enpen 6	1.9	3
TriCurin 7	0.5	6
T-statistic	-1.2857	
p-value	0.2395	

IC50: 6 ±1.9 μM for Tricurin in Enpen; IC50: 7±0.5 μM for Tricurin only;

References

S1. Straub, E., Dreer, M., Fertey, J., Iftner, T. & Stubenrauch, F. The viral E8^E2C repressor limits productive replication of human papillomavirus 16. *J. Virol*. **88**, 937–947 (2014).

S2. Egawa, N., Wang, Q., Griffin, H. M., Murakami, I., Jackson, D., Mahmood, R. et al. HPV16 and 18 genome amplification show different E4-dependence, with 16E4 enhancing E1 nuclear accumulation and replicative efficiency via its cell cycle arrest and kinase activation functions. *PLoS Pathog.*. **13**, e1006282 (2017).

S3. Al-Haj, Latifa, Blackshear, PerryJ. & Khabar, KhalidS. A. Regulation of p21/CIP1/WAF-1 mediated cell-cycle arrest by RNase L and tristetraprolin, and involvement of AU-rich elements. *Nucleic Acids Res*. **40**, 7739–7752 (2012).
